# 

**Published:** 1871-05

**Authors:** 


PAGE.
A Case of Supposed Poisoning from Daily Contact with the Gases Generated by the Combustion of Gunpowder. By E. D. WITHERS, M. D., Atlanta, Ga. . . . . 65
Acute Rheumatism. By W. M. WELLS, M. D., Atlanta, Ga. . . . . 70
Rupture of the Spleen, Discharge through Alimentary Canal, and Recovery. By WM. ABRAM LOVE, M. D., Atlanta, Ga. . . . . 75
Extracts from Minutes of Atlanta Academy of Medicine . . . . . 79
Clinique of Atlanta Medical College. Service of Dr. J. G. WESTMORELAND . . . . . 82

SELECTIONS.

On the Action of Light in Small-Pox. By J. H. WATERS, M.B., M.C., &c. 85

On Hepatic Fistulæ By M. SIGNEROLIES . . . . . 88

Case of Acute Renal Dropsy. Under the care of Dr. HENRY THOMPSON.. 90

On the Production of Hæmorrhage, Anæmia, Œdema, and Emphysema in the Lungs by Injuries to the Base of the Brain. By C. E. BROWN-SEQUARD, M. D., &c . . . . . 92

Notes on Laminaria Tents By J. C. NOTT, M. D., New York . . . . . 94

Hospital for Sick Children. Notes of a Clinical Lecture on Chorea. By By DR. DICKINSON . . . . . 98

Hydrate of Chloral in Singultus. By T. L. LEAVITT, M. D., of Germantown, Pa . . . . . 101

Tubercular Pneumonia. By D. FRANCIS CONDIE, M. D., of Philadelphia. 102

Transfusion Successful in a Case of Post-partum Hemorrhage. . . . . 106

EDITORIAL.

Atlanta Medical College . . . . . 108
A Statement of Facts in Regard to the Controversy Between the Late Faculty of the Atlanta Medical College and Certain Members of the Georgia Medical Association . . . . . 109
Georgia Medical Association—An Abstract of the Proceedings. . . . . 121
Striking Features of the Prosecution of the Atlanta Academy of Medicine . . . . . 128
Extract from the Records of the Atlanta Academy of Medicine, April 22, 1871 . . . . . 128

